# Clay mineralogical and geochemical proxies of the East Asian summer monsoon evolution in the South China Sea during Late Quaternary

**DOI:** 10.1038/srep42083

**Published:** 2017-02-08

**Authors:** Quan Chen, Zhifei Liu, Catherine Kissel

**Affiliations:** 1 State Key Laboratory of Marine Geology, Tongji University, Shanghai, China; 2 Laboratoire des Sciences du Climat et de l’Environnement/IPSL, CEA-CNRS-UVSQ, Université Paris-Saclay, Gif-sur-Yvette, France

## Abstract

The East Asian summer monsoon controls the climatic regime of an extended region through temperature and precipitation changes. As the East Asian summer monsoon is primarily driven by the northern hemisphere summer insolation, such meteorological variables are expected to significantly change on the orbital timescale, influencing the composition of terrestrial sediments in terms of both mineralogy and geochemistry. Here we present clay mineralogy and major element composition of Core MD12-3432 retrieved from the northern South China Sea, and we investigate their relationship with the East Asian summer monsoon evolution over the last 400 ka. The variability of smectite/(illite + chlorite) ratio presents a predominant precession periodicity, synchronous with the northern hemisphere summer insolation changes and therefore with that of the East Asian summer monsoon. Variations in K_2_O/Al_2_O_3_ are characterized by eccentricity cycles, increasing during interglacials when the East Asian summer monsoon is enhanced. Based on the knowledge of sediment provenances, we suggest that these two proxies in the South China Sea are linked to the East Asian summer monsoon evolution with different mechanisms, which are (1) contemporaneous chemical weathering intensity in Luzon for smectite/(illite + chlorite) ratio and (2) river denudation intensity for K_2_O/Al_2_O_3_ ratio of bulk sediment.

The East Asian monsoon is a major component of the modern climate system^1^. It controls the environmental conditions prevailing over extended regions through seasonal changes of wind and precipitation^2^,^3^. As the monsoon system is primarily driven by the solar insolation received at the Earth’s surface, in turn related to the changing Earth orbital parameters, its intensity and impact are variable on orbital timescales. Because information of monsoonal meteorological variables (temperature, precipitation, and wind stress) is not directly preserved in geological archives, many investigations have been carried out to reconstruct the variability of the East Asian monsoon with proxies that reveal the influence of monsoonal climatic changes on the physical, chemical, and biological processes on land and in ocean^4^,^5^,^6^,^7^.

Among the continental sequences, Chinese loess sequence is a unique archive because it records the variability of both wind strength and aridity. Grain-size of Chinese loess, generally increasing during glacials^8^,^9^,^10^,^11^, is interpreted as an indicator of the East Asian winter monsoon (EAWM) intensity. Magnetic susceptibility records from Chinese loess indicate periodical intensifications of the East Asian summer monsoon (EASM) in concert with the northern hemisphere summer insolation (NHSI) on both eccentricity and precession bands^12^,^13^,^14^,^15^. Meanwhile, oxygen isotope records from stalagmites in East and South China are considered as a proxy of the monsoonal precipitation intensity, the variations of which strongly support the hypothesis that the EASM is driven by NHSI changes on precession band^16^,^17^,^18^.

The marine sediment sequences provide alternatives to study the past variability of the East Asian monsoon. Proxies based on biogenetic materials such as foraminifera, coccolithophore, and radiolarian were developed to reconstruct the history of primary productivity and sea surface temperature and to reveal the evolution of the EASM on the orbital and suborbital timescales^19^,^20^,^21^,^22^. Terrigenous clastic particles in marine sediments also document the climate changes related to the EASM evolution. Clay mineral is one of the most frequently used tools to reconstruct paleoclimatic changes on different timescales. For instance, clay mineralogical investigations in the Indian Ocean revealed orbital signals in illite/smectite ratio and the palygorskite/illite ratio, which were used to reconstruct the monsoon strength^23^,^24^. Such investigations were also performed on sedimentary sequences retrieved from the South China Sea (SCS). Glacial-interglacial cycles were observed with high smectite/(illite + chlorite) ratio during interglacial periods^25^,^26^,^27^. The underlying mechanism was proposed to be either the surface circulation related to monsoon winds^26^ or the balance of chemical weathering versus physical erosion on land^25^. However, high-resolution clay mineral records do not always present such glacial-interglacial cyclicity^28^,^29^. Indeed, a predominant control by precession is observed in the variations of smectite/(illite + chlorite) ratio at ODP Site 1145, which is probably induced by changes in weathering and transport^28^. The clay mineralogical record since the last glacial maximum revealed the complexity of terrigenous input from surrounding drainage systems and transport processes^29^. The mechanism that links the EASM evolution and the sediment composition remains discussible.

In order to better understand the relationship between the terrigenous sediment composition and the EASM evolution, and what the driving mechanism is, we investigated the clay mineral assemblage and major element composition in Core MD12-3432 that was retrieved from the northern SCS (Fig. 1).

## Results

The clay mineral assemblage of Core MD12-3432 mainly consists of smectite (23–59%) and illite (22–43%) with minor chlorite (13–27%) and kaolinite (4–13%) (Fig. 2). The smectite record presents frequent and significant short-term variations, coeval and opposite to those of illite and chlorite. Kaolinite content shows a glacial-interglacial variability with relatively higher values during glacial periods compared to interglacials. The illite crystallinity varies between 0.13° and 0.18° Δ2θ (Fig. 2).

The knowledge of sediment provenances is the basis for deciphering the climatic information preserved in the clay mineral assemblage. Detrital material deposited in the northern SCS has been shown to mainly derive from the Pearl River drainage area, Taiwan, and Luzon^30^. Due to the different geological and tectonic settings prevailing in each region, clay mineral species are distinct (Fig. 3) and they remain constant during the Late Quaternary^26^,^29^,^31^,^32^. The Pearl River drainage area has stable tectonic regime, flat topography, and warm/humid monsoonal climate. Rocks and soils in the Pearl River catchment experience strong and sustained chemical weathering with relatively weak physical erosion. These conditions favour the formation and accumulation of kaolinitic soils that may take more than 1 Ma^31^,^33^. In Taiwan, the river denudation is intense because of active tectonic, steep topography, and heavy monsoon/typhoon rainfall^34^,^35^,^36^,^37^. Sedimentary and metamorphic bedrocks in Taiwan are rapidly eroded. This strong physical erosion inhibits chemical weathering and fosters the formation and delivery of primary minerals of illite and chlorite^38^,^39^. Given the similarity of tectonic and climatic settings in Taiwan and Luzon, the strong monsoonal rainfall may also result in intense river denudation in Luzon. However, Luzon is characterized by widely spread volcanic rocks, which can be rapidly weathered to form abundant smectite^32^,^40^,^41^,^42^,^43^. The strong denudation would therefore promote the erosion and delivery of smectite, preventing further weathering and production of kaolinite to occur^32^,^40^,^44^. Although human behaviour might alter the clay mineral assemblage in river samples^45^, this anthropogenic influence is minor and the clay minerals characterizing these three sources remain constant over the late Quaternary because of the unique geological and tectonic settings in these sources. Modern clay mineral assemblages in these sources are, therefore, appropriate references for provenance analysis.

The clay mineral assemblage of Core MD12-3432 is reported in a ternary diagram where end-members are illite + chlorite, kaolinite, and smectite. The regions associated to each end-member are determined on the basis of the studies of continental sediments from the different potential source areas^23^,^25^,^31^. The clay mineral assemblage of Core MD12-3432 is linearly distributed between end-members of illite + chlorite and smectite with higher kaolinite contents during glacial periods (Fig. 3), indicating a mixture of clay minerals from multiple sources: all smectite derives from Luzon, all kaolinite sources from the Pearl River, and illite and chlorite may originate from Taiwan and the Pearl River. Illite and chlorite are both primary minerals and they have a similar distribution in the SCS^26^,^29^,^40^. Thus, illite provenance analysis is also relevant to chlorite provenance. Although the ternary diagram cannot discriminate Taiwan and Pearl River provenances, illite from the Pearl River is well weathered with low crystallinity (0.19°–0.24°Δ2θ), while illite from Taiwan is formed directly from strong physical erosion with high crystallinity (0.12°–0.18°Δ2θ)^31^,^38^. The well crystallized illite of Core MD12-3432 (0.13°–0.18° Δ2θ) indicates Taiwan as the main contributor of illite and chlorite.

Smectite, illite and chlorite are the major components of the clay mineral assemblage in Core MD12-3432. Because they originate from different sources, the smectite/(illite + chlorite) ratio reflects the relative contribution of clay minerals from Luzon versus Taiwan^29^. As we discussed above, the supply of illite and chlorite from Taiwan results from the rainfall-driven erosion, and the supply of smectite from Luzon depends on both rapid chemical weathering and physical erosion. Smectite/(illite + chlorite) therefore constitutes an indicator of contemporaneous chemical weathering intensity on land. Regarding of the time series variations, the most prominent feature of smectite/(illite + chlorite) ratio of Core MD12-3432 is the short-term fluctuations, which are coeval with the previously reported smectite/(illite + chlorite) variations at ODP Site 1145^28^ (Fig. 4a,b). The comparison with the clay mineral record at ODP Site 1146 is difficult due to the low resolution^26^, but we still observe some simultaneous peaks in Core MD12-3432 and ODP Site 1146 (Fig. 4a,c). In addition to these short-term fluctuations, smectite/(illite + chlorite) ratio of Core MD12-3432 generally increases during interglacial periods (Fig. 4a). This subtle glacial-interglacial cyclicity has also been reported at ODP Site 1146^26^ (Fig. 4c). Redfit spectrum analyses^46^ were applied to the smectite/(illite + chlorite) profiles at all three sites. A dominant precession periodicity (23 kyr) is observed at Core MD12-3432 and ODP Site 1145, while eccentricity signals are observed at all three sites and dominate the smectite/(illite + chlorite) variations at ODP Site 1146 due to the low resolution (Fig. 5a–c). These results suggest that the orbital forcing exerts a major influence on this ratio in this study area. The synchronicity of maxima in the smectite/(illite + chlorite) ratio and the NHSI (Fig. 4a–c and g) suggests that changes in chemical weathering intensity of the clay fraction in Luzon is related to the NHSI. The NHSI is the driving force of the EASM^17^, which controls the climate regime in the SCS region^47^. The strong EASM is accompanied by warm and humid climate, favouring the rapid chemical weathering of volcanic rocks in Luzon^41^,^48^. Therefore, the simultaneous variations in the smectite/(illite + chlorite) ratio and the NHSI indicate that the NHSI-driven EASM intensity plays a dominant role in controlling chemical weathering intensity in Luzon as revealed by the smectite/(illite + chlorite) ratio of marine sediments. Smectite/(illite + chlorite) ratio can, hence, act as a proxy of the EASM intensity.

Kaolinite is a minor component in the clay mineral assemblage in Core MD12-3432 (Fig. 2). As a weathering product, kaolinite has usually been used as an indicator of chemical weathering intensity on the tectonic timescale^5^. In Core MD12-3432, variations in kaolinite content are observed at glacial/interglacial timescale, which is much shorter than tectonic timescale, with high contents during cold and dry glacial periods (Fig. 4d and f). This observation does not fit with the understanding that chemical weathering giving rise to kaolinite content is fostered by warm/humid climate^41^,^48^. Indeed, the supply of kaolinite from the Pearl River reflects the intensity of physical erosion rather than chemical weathering in the drainage area because the abundant kaolinite in the Pearl River are formed by long-term weathering and accumulation^31^. Furthermore, the transport in ocean is also a key process controlling the clay mineral assemblage of marine sediments^49^. Sea level changes seem to be the mechanism underlying the kaolinite variation pattern of Core MD12-3432. Due to the presence of largely extended shelf between the Pearl River mouth and our site, the lowering of sea level during glacials would enhance erosion by inducing river incision on the exposed shelf and notably reduce the transport distance of kaolinite from the Pearl River. Meanwhile, sea level changes have much weaker impacts on the other two provenances because the shelves off Taiwan and Luzon are narrow. Consequently, the low sea level during glacial periods increases the relative contents of kaolinite from the Pearl River (Figs 3 and 4d).

Major element composition offers an alternative to investigate the physical and chemical conditions of terrigenous sediments. Due to the relative contribution of biogenic/terrigenous materials, all terrigenous elements show the same variation pattern opposite to that of CaO% (see Supplementary Fig. S1 and Text S1). In order to examine the changes within terrigenous fraction, we focus on K_2_O/Al_2_O_3_ ratio. Indeed, because Al is resistant to leaching while K is more mobile during chemical weathering, K_2_O/Al_2_O_3_ ratio can trace the chemical weathering degree of bulk sediments^5^,^50^,^51^. Although grain-size effect may challenge the reliability of element ratio proxies, it does not control the K_2_O/Al_2_O_3_ ratio in Core MD12-3432 because K is preferably found in illite while Al is a major component of all clay minerals^52^,^53^,^54^. Therefore, an increase in K_2_O/Al_2_O_3_ ratio would indicate a decrease in chemical weathering degree of bulk sediments. The variations in K_2_O/Al_2_O_3_ ratio of Core MD12-3432 present a glacial-interglacial cyclicity (Fig. 4e). This is confirmed by the spectral analysis that shows a predominant eccentricity periodicity (Fig. 5d). However, the periodic increase of this ratio occurs during interglacials (Fig. 4e), in phase with the increase in the chemical weathering as illustrated by smectite/(illite + chlorite) ratio (Fig. 4a). This apparent contradiction indicates that the increase of K_2_O/Al_2_O_3_ ratio does not correspond to contemporaneous weak chemical weathering at the source areas. Changes in source areas may be responsible for the apparent out-of-phase increases^53^,^55^. Due to strong physical erosion and weak chemical weathering, K_2_O/Al_2_O_3_ ratio of sediments in Taiwan is higher than that in Luzon and the Pearl River drainage area^31^,^32^,^38^,^39^,^56^. Given the fact that Taiwan exports the largest amount of suspended sediments among all three provenances^34^,^35^,^36^,^37^, the intensified EASM rainfall tends to preferentially enhance the river denudation rate in Taiwan and, thus, increases the relative contribution of erosive detrital material with high K_2_O/Al_2_O_3_ ratio from Taiwan. Therefore, rather than contemporaneous chemical weathering on land, the increase of K_2_O/Al_2_O_3_ ratio in bulk sediments of Core MD12-3432 reflects strong river denudation that is fostered by intensifications of the EASM precipitation on eccentricity band.

## Discussion and Summary

The EASM largely controls the climate regime prevailing in the SCS region^47^, bringing warm/humid climate with heavy precipitation in summer. Such climate features foster both chemical weathering and river denudation on land. As the three sediment sources of terrigenous sediments in the northern SCS are characterized by distinct geological and tectonic settings, their sediment supplies respond in different ways to such chemical and physical processes driven by the EASM changes on orbital timescales. Our clay mineralogical and geochemical investigations on Core MD12-3432 sediments support the hypotheses that the chemical weathering intensity of volcanic rocks in Luzon is sensitive to the EASM induced warm/humid climatic changes, the river denudation is reactive to the EASM precipitation in Taiwan, and the weathering in the Pearl River drainage area does not respond to the EASM variations on orbital timescale.

Based on the knowledge of the different characteristics of each sediment source area, our results reveal the mechanism underlying the relationship between variations in the EASM intensity and the smectite/(illite + chlorite) and K_2_O/Al_2_O_3_ ratios of sediments in the northern SCS. Smectite/(illite + chlorite) constitutes a tracer of contemporaneous chemical weathering intensity in Luzon, the variability of which is dominated by the EASM induced variations in temperature and moisture. K_2_O/Al_2_O_3_ ratio reveals the glacial-interglacial cyclicity of the river denudation intensity controlled by the EASM precipitation. In addition, it is obvious that kaolinite content is not an appropriate indicator of EASM on the orbital timescale because it is primarily controlled by sea level changes rather than contemporaneous chemical weathering intensity in the source area.

## Methods

**Figure 1:**
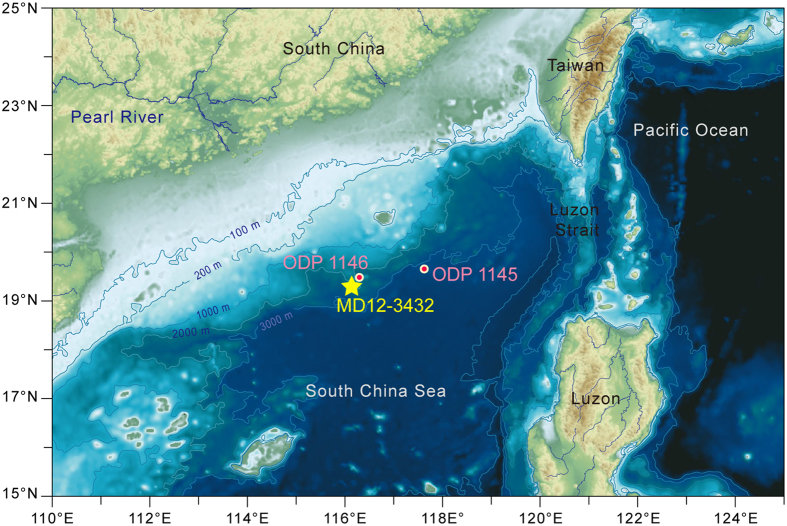
Topography of the northern South China Sea. The map is generated from the ETOPO1 global relief model^64^ (http://www.ngdc.noaa.gov/mgg/global/global.html) using Global Mapper (version 13 by Blue Marble Geographics^®^, http://www.bluemarblegeo.com/products/global-mapper.php). The locations of Core MD12-3432 (star) and ODP Sites 1145 and 1146 (dots) are presented.

**Figure 2:**
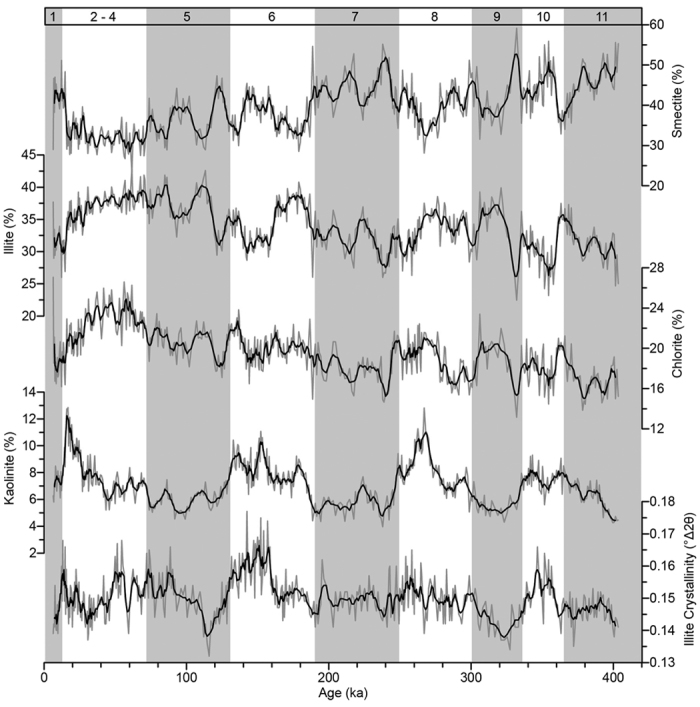
Variations in clay mineral assemblage of Core MD12-3432. Grey bars highlight interglacial periods with marine isotope stages (MIS) noted on top.

**Figure 3:**
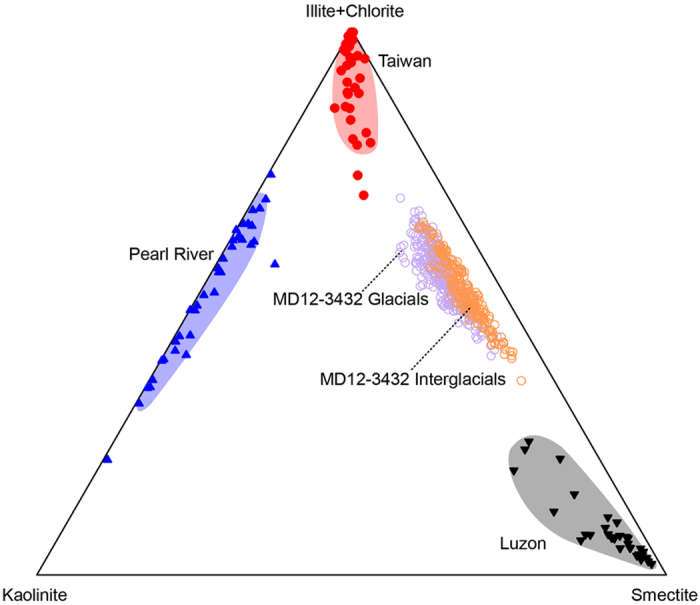
Ternary diagram of clay mineral assemblages. The clay mineral assemblage of Core MD12-3432 is compared to those of surface sediments from potential provenances, *i.e*. Taiwan^38^, Pearl River^31^, and Luzon^32^.

**Figure 4:**
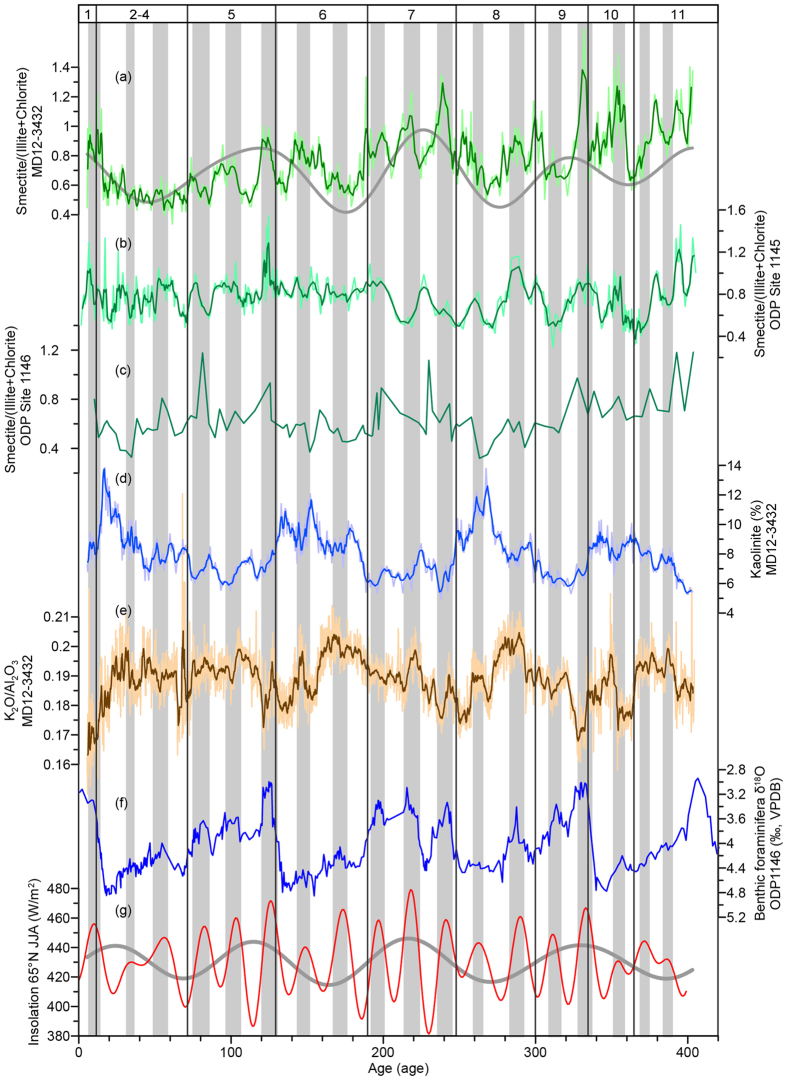
Time-series variations in clay mineral and major element ratios, which are (**a**) smectite/(illite + chlorite) of Core MD12-3432 with the 100-ka filtered curve (grey dashed line), (**b**) smectite/(illite + chlorite) at ODP Site 1145^28^, (**c**) smectite/(illite + chlorite) at ODP Site 1146^26^, (**d**) kaolinite content of Core MD12-3432, (**e**) K_2_O/Al_2_O_3_ ratio of Core MD12-3432, (**f**) benthic foraminifera δ^18^O at ODP Site 1146^62^,^63^, and (**g**) mean summer insolation at latitude of 65°N^65^ with the 100-ka filtered curve (grey dashed line). Vertical lines mark the glacial/interglacial boundaries with marine isotopic stage number noted on top, and shaded areas highlight the intervals of maximum insolation. The age model of ODP Site 1145 was correlated to the same ODP Site 1146 chronological framework as used for Core MD12-3432.

**Figure 5:**
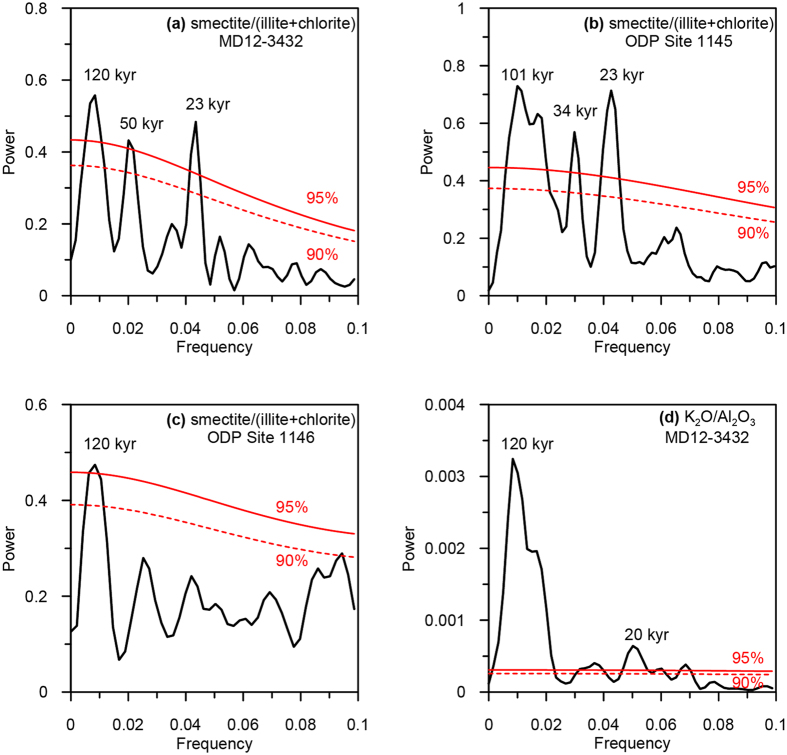
Redfit spectral analyses of smectite/(illite + chlorite) and K_2_O/Al_2_O_3_ ratios using PAST software^46^. Spectral analyses of smectite/(illite + chlorite) at (**a**) Core MD12-3432, (**b**) ODP Site 1145 and (**c**) ODP Site 1146, and (**d**) K_2_O/Al_2_O_3_ ratio of Core MD12-3432 are presented. The false-alarm level of 95% and 90% are illustrated by red solid lines and red dash lines, respectively. The characterizing periodicities are noted together with the spectrums.

Investigations of clay mineral assemblage and major element composition were conducted on Core MD12-3432 (19°16.88′N, 116°14.52′E, 2125 m water depth), which was retrieved from the northern SCS in 2012 with R.V. *Marion Dufresne* during the French-Chinese CIRCEA cruise organized within the LIA-MONOCL framework^57^. The core is 50.8 m long and it is located on the lower continental slope of the northern SCS (Fig. 1). The sediment lithology is homogenously dominated by grey clays^57^. All measurements presented in this study were performed at the State Key Laboratory of Marine Geology, Tongji University.

### Clay mineralogical investigation

A total of 502 samples with a depth-resolution of 10 cm were taken for clay mineralogical analysis. Following previously published studies, oriented mounts were prepared, and clay minerals were identified by X-ray diffraction (XRD) on these oriented mounts of non-calcareous clay-sized (<2 μm) particles^25^,^26^,^58^. The analysis was performed under three conditions, *i.e*., air-drying, ethylene-glycol solvation for 24 h, and heating at 490 °C for 2 h, using a PANalytical X’Pert Pro diffractometer with CuKα radiation and Ni filter, under a voltage of 45 kV and an intensity of 40 mA.

Identification of clay minerals and calculation of their relative proportions were made according to the position and area of the (001) series of basal reflections on the three XRD diagrams, *i.e*., smectite (001) including illite/smectite mixed-layers at 17 Å, illite (001) at 10 Å, and kaolinite (001) and chlorite (002) at 7 Å^25^,^26^,^58^. Relative proportions of kaolinite and chlorite were determined based on the ratio from the 3.57/3.54 Å peak areas. Additionally, illite crystallinity was obtained from the half height width of the 10 Å peak^40^,^59^. Semi-quantitative calculations of all the included peak parameters were performed on the glycolated curves using MacDiff software^60^.

### Analysis of major element composition

Major element data of Core MD12-3432 were previously investigated^61^ with XRF core-scanner and wavelength-dispersive XRF. We corrected the influence of water content changes on XRF core-scanning element intensities and calibrated these semi-quantitative data to oxide concentrations using the normalized polynomial scaled calibration method^61^.

### Establishment of age model

A composite age model of the nearby ODP Site 1146 was firstly constructed by combining two published age models. The age model between 0 and 350 ka was established using the planktonic oxygen isotope record refereed to those of Chinese speleothem^62^. The remaining part at the bottom of the core was established by astronomical tuning of benthic oxygen isotope record^63^. This composite chronological framework was transferred to Core MD12-3432 using the very similar carbonate content records with a total of 27 correlation points (see Supplementary Fig. S2). Core MD12-3432 covers the last 400 ka with a sedimentation rate varying between 4.5 and 23.8 cm/ky.

## Additional Information

**How to cite this article**: Chen, Q. *et al*. Clay mineralogical and geochemical proxies of the East Asian summer monsoon evolution in the South China Sea during Late Quaternary. *Sci. Rep.* **7**, 42083; doi: 10.1038/srep42083 (2017).

**Publisher's note:** Springer Nature remains neutral with regard to jurisdictional claims in published maps and institutional affiliations.

## Supplementary Material

Supplementary Information
