# Supplementary material for: Clay mineralogical and geochemical proxies of the East Asian summer monsoon evolution in the South China Sea during Late Quaternary
Source: Sci Rep. 2017 Feb 8;7:42083. doi: 10.1038/srep42083 (PMC5296726; doi:10.1038/srep42083)
Supplement: Supplementary Information [file srep42083-s1.pdf]

Supplementary Information

Clay mineralogical and geochemical proxies of the East Asian summer monsoon evolution in the South China Sea during Late Quaternary

Quan Chen[1,2,*], Zhifei Liu[1], Catherine Kissel[2]

1. State Key Laboratory of Marine Geology, Tongji University, Shanghai, China
2. Laboratoire des Sciences du Climat et de l'Environnement/IPSL, CEA-CNRS-UVSQ, Université Paris-Saclay, Gif-sur-Yvette, France

* Correspondence and requests for materials should be addressed to Q.C. (email: quan.chen@hotmail.com)

Text S1 Major Element Composition

In Core MD12-3432, major elements of bulk sediment are dominated by SiO_2 (29%-59%), followed by CaO (4%-25%) and Al_2O_3 (10%-17%), and minor content of Fe_2O_3 (4%-7%), K_2O (2%-4%), and TiO_2 (0.4%-0.7%). All terrigenous element oxides exhibit the same variation pattern, increasing during glacial periods (Supplementary Fig. S2). On the contrary, CaO% decreases notably during glacials (Supplementary Fig. S2). Short-term variations are also observed with the same opposed pattern between terrigenous element oxides and CaO (Supplementary Fig. S2).

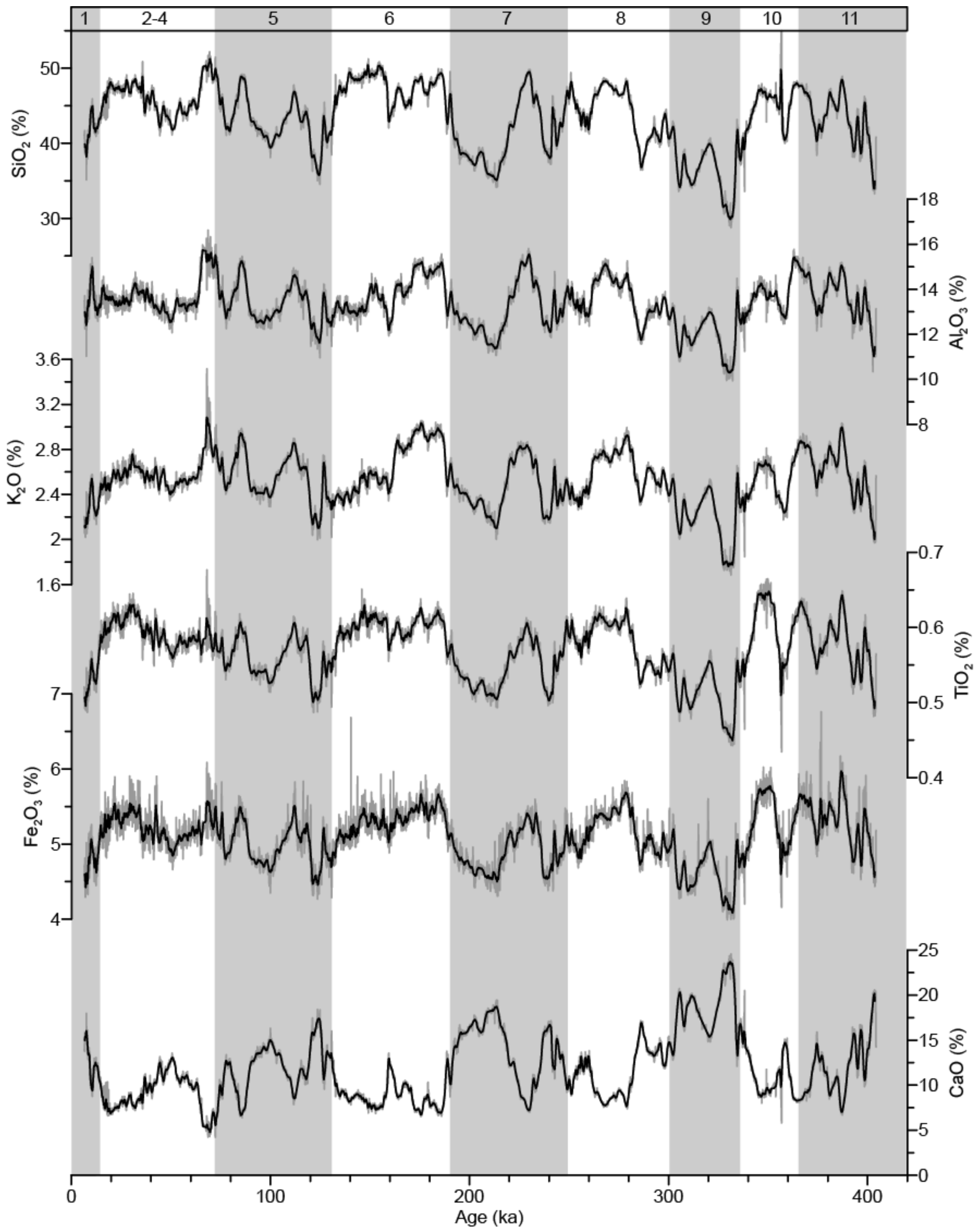

Figure S1 Composition of major element oxides calibrated from XRF-scanning data[1]. Shaded areas highlight interglacial periods.

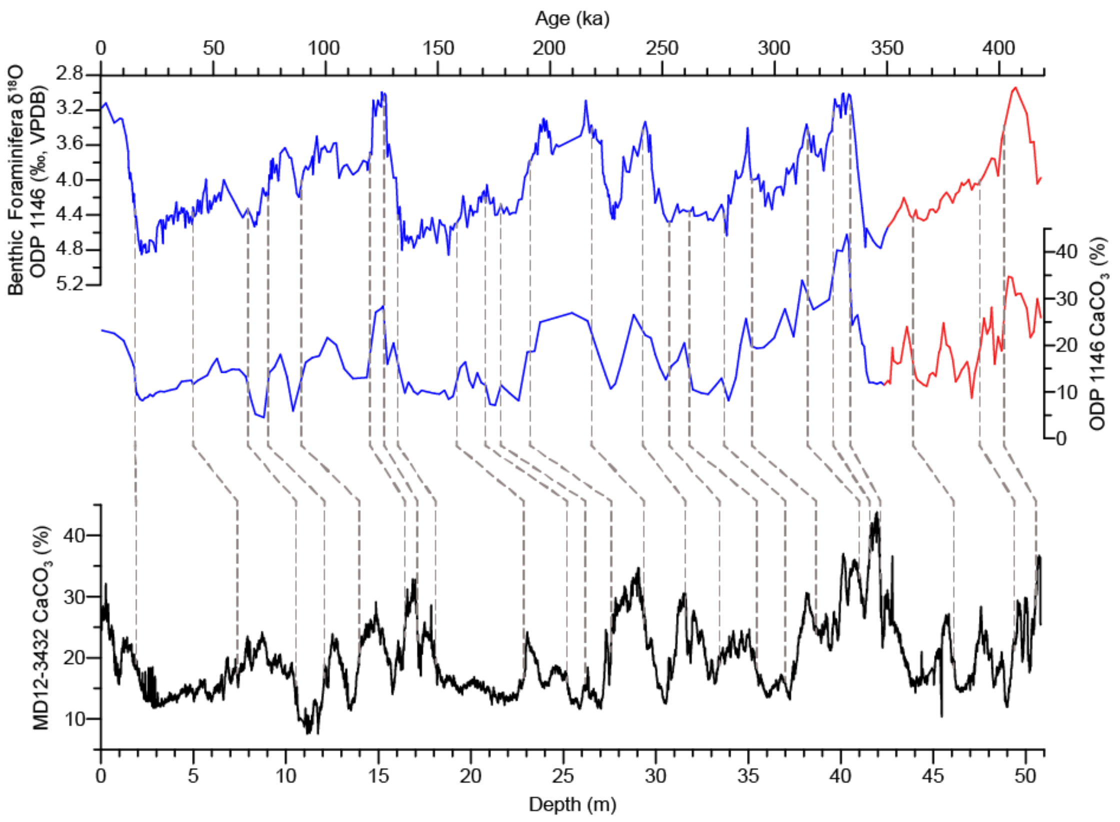

Figure S2 Age model of Core MD12-3432. Carbonate content calibrated from XRF core-scanning data is displayed versus depth, together with the age-based $CaCO_3$% profile and benthic foraminifera $\delta^{18}O$ record form ODP Site 1146[2,3]. As a composite age model of ODP Site 1146 was used, the blue and red curves represent age models reported by Caballero-Gill et al[2]. and Clemens et al.[3], respectively. Grey dashed lines illustrate the position of pair points.

Reference

1 Chen, Q., Kissel, C., Govin, A., Liu, Z. & Xie, X. Correction of interstitial water changes in calibration methods applied to XRF core-scanning major elements in long sediment cores: Case study from the South China Sea. *Geochemistry, Geophysics, Geosystems* **17**, 1925-1934, doi:10.1002/2016GC006320 (2016).

2 Caballero-Gill, R. P., Clemens, S. C. & Prell, W. L. Direct correlation of Chinese speleothem δ18O and South China Sea planktonic δ18O: Transferring a speleothem chronology to the benthic marine chronology. *Paleoceanography* **27**, doi:10.1029/2011PA002268 (2012).

3 Clemens, S. C., Prell, W. L., Sun, Y., Liu, Z. & Chen, G. Southern Hemisphere forcing of Pliocene δ18O and the evolution of Indo-Asian monsoons. *Paleoceanography* **23**, PA4210, doi:10.1029/2008PA001638 (2008).
